# Variant Superficial Branch of Radial Artery along with Supplementary Tendons on the Dorsum of the Hand and Their Surgical Implications

**DOI:** 10.1155/2016/9581759

**Published:** 2016-10-05

**Authors:** Satheesha B. Nayak, Naveen Kumar, Ashwini Aithal Padur, Surekha D. Shetty

**Affiliations:** Department of Anatomy, Melaka Manipal Medical College, Manipal University, Manipal Campus, Manipal 576104, India

## Abstract

Variations of radial artery, in both its course and branching pattern in the anatomical snuffbox, are clinically significant for the plastic surgeons, cardiologists, and radiologists. Reports on its abnormal high origin and subsequent superficial course have been well documented. Herein, we report an unusual superficial branch of the radial artery given off before its entry into the palm by passing between the two heads of first dorsal interosseous. It eventually divided into princeps pollicis and radialis indicis arteries at the first web space of palm as a unique vascular variation. Apart from this, in the present case, the tendon of extensor digiti minimi and of extensor indicis divided into two parts. The split tendons of extensor digiti minimi were inserted to the dorsal digital expansion of the digitus minimus. However, lateral tendon of split extensor indicis was inserted along with the tendon of extensor digitorum to the index finger and the medial one was inserted along with the tendon of extensor digitorum to the middle finger. Unusual superficial branch of radial artery on the dorsum of the hand is vulnerable for an iatrogenic injury during surgical approaches in the region. Supplementary extensor tendons on the hand are one of the potential causes for the tenosynovitis.

## 1. Introduction

Accurate and detailed knowledge of the relationships and possible anatomical variations of the branching pattern of radial artery is vital during reparative surgery in this region. In addition, iatrogenic trauma due to occurrence of superficial branches may lead to a life-threatening hemorrhage. Inadequate knowledge of the anatomical variations of the arterial pattern may make surgery difficult.

Radial artery is the terminal branch of the brachial artery given off in the cubital fossa. Following its origin, it assumes superficially downward course to the wrist along the radial side of the forearm. At the distal part of the forearm it winds around the lateral side of forearm after giving superficial palmar branch and enters into the anatomical snuff box, before reaching proximal end of the first interosseous space where it passes between the two heads of first dorsal interosseous muscle and between two heads of adductor pollicis to form the deep palmar arch of the palm [1].

In the anatomical snuff box it gives dorsal carpal branch for the formation of dorsal carpal arch. Before its entry to the palm, on the dorsum of the hand it gives slender branches to the lateral side of the dorsum of the thumb and first dorsal metacarpal artery. In the palm it generally gives princeps pollicis artery and radialis indicis branches before it contributes to deep palmar arch.

Normally, the extensor muscles have single tendon on the dorsum of hand except for extensor digitorum. The tendon of extensor digitorum divides distally into 4 tendons which pass in a common synovial sheath together with tendon of extensor indicis and diverge on the dorsum of the hand to get inserted into the medial four digits [2].

The extensor tendons of the hand are distally interconnected by oblique interconnections known as juncturae tendinum. Knowledge of detailed pattern of the tendons and their oblique interconnections is important in hand assessment and other reconstructive procedures [3].

Combined variations of radial artery and extensor tendons are rare. We discuss the clinical importance of concurrent variations of radial artery and the extensor tendons in this report. Anatomical knowledge on variant branching pattern of radial artery as well as abnormal tendon disposition on the dorsum of the hand is of considerable importance during several clinical approaches and therapeutic practices.

## 2. Case Report

**Figure 1:**
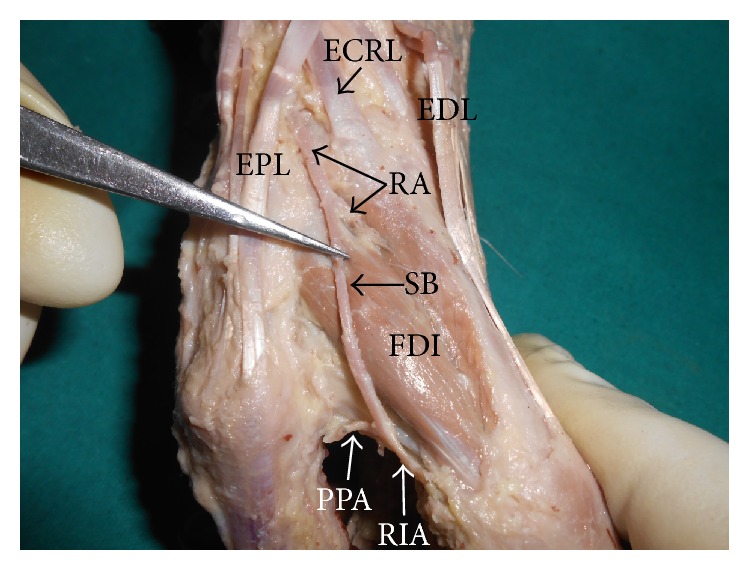
Lateral view of the dorsum of hand showing the superficial branch (SB) of radial artery (RA). (EDL: extensor digitorum; ECRL: extensor carpi radialis longus; EPL: extensor pollicis longus; FDI: first dorsal interosseous; PPA: princeps pollicis artery; RIA: radialis indicis artery).

During the dissection of the left upper limb of an adult male cadaver aged approximately 70 years, we found concurrent variations of radial artery and tendons of long extensors of the digits. The radial artery, after passing through the anatomical snuff box, gave a large superficial branch before continuing into the palm by passing between the two heads of first dorsal interosseous. The superficial branch ran distally over the first dorsal interosseous and terminated by dividing into princeps pollicis and radialis indicis arteries at the first web space [Figure 1]. This variant superficial branch and its terminal branches were just covered only by skin and superficial fascia and were thus very vulnerable.

**Figure 2:**
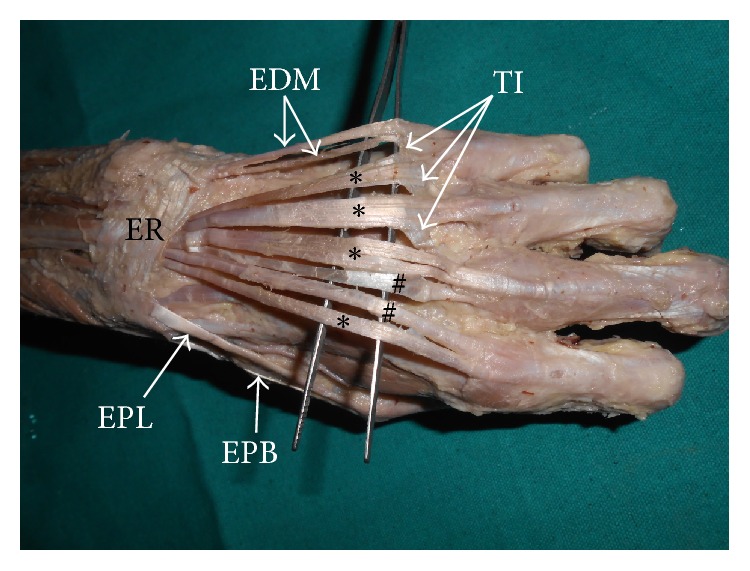
Dissection of dorsum of hand and wrist showing the additional tendons. (ER: extensor retinaculum; EDM: extensor digitorum; EPL: extensor pollicis longus; EPB: extensor pollicis brevis; TI: tendinous interconnections; *∗*: tendons of extensor digitorum; #: tendons of extensor indicis).

The extensor digitorum divided into four tendons, which had normal insertion as described in the Anatomy textbooks. Tendon of extensor digiti minimi divided into two equal parts as it passed deep to the extensor retinaculum. Both the tendons were inserted to the dorsal digital expansion of the digitus minimus. Tendon of extensor indicis divided into two parts. The lateral part was inserted along with the tendon of extensor digitorum to the index finger and the medial one was inserted along with the tendon of extensor digitorum to the middle finger. The tendons going to the medial three digits were connected to each other through broad tendinous interconnections [Figure 2].

## 3. Discussion

Awareness of radial arterial variation in its origin and branching pattern has great importance in various clinical fields and basic medical studies. High origin of radial artery from brachial artery is one among its frequent anatomical variations as its prevalence is reported to be 14.26% in cadaveric studies and 9.75% in angiographic studies [4]. Reports on its variant origin and in its proximal course are quite common compared to its distal course and termination [5]. Higher origin of radial artery associated with its complete superficial course, termed as superficial brachioradial artery, has been reported by Sharmila Bhanu et al. [6]. An extremely superficial course of radial artery in the anatomical snuff box has been reported by Jyothsna et al. [5]. However, an occurrence of anomalous superficial branch of radial artery at the anatomical snuff box region of the hand is seldom reported.

The superficial arteries often pose iatrogenic traumatic complications as they are mistaken for veins during intravenous injections [7, 8]. Awareness of superficial arteries is also important to the plastic surgeons particularly during elevation of forearm flaps [9, 10]. Presence of superficial arteries might often lead to misinterpretation in contrast radiographic procedures.

Extensor muscles of the forearm have a relatively consistent architecture. However, at times they may have prominent anatomic variations in their tendons, particularly on the ulnar side of the hand [11]. Tanaka et al. have undertaken an extensive anatomic study of the 5th extensor tendon compartment and extensor digiti minimi tendon to evaluate its contribution to the development of tenosynovitis and limit the usefulness of the extensor digiti minimi (EDM) for tendon transfer [12].

EDM tendon triggering is a less common disease when compared with other tendons triggering. Its impingement on the extensor retinaculum aggravates the condition into double triggering [13]. Zilber and Oberlin, in their anatomical study on variant extensor tendons to the fingers on the dorsum of the hand, reported the exhibition of single tendon of extensor indicis in all the specimens. However, they did report some of the rare observations like no tendon contribution to little finger form extensor digitorum, absence of extensor indicis bilaterally, and tendon slip from the extensor digiti minimi to the ring finger [14].

Kocabiyik et al. reported bilateral extensor tendon variations which included tripled tendon for middle finger from the extensor digitorum, double tendons to ring finger, and extensor digiti minimi having duplicate tendon with an abnormal communicating tendon between extensor digitorum tendon to ring finger and extensor digiti minimi muscle tendons. While these variant tendon dispositions were observed in one hand, on the other hand abductor pollicis tendon itself trifurcated and one of these tendons also attached to tendon of abductor pollicis brevis as an additional slip [11].

Seradge et al. reported EDM in which two tendon slips attached to the little finger and one to the ring finger's metacarpophalangeal joint [15]. Extensor digitorum is known to have multiple tendons to middle and ring fingers and its tendon contribution to ring finger often may bifurcate. However, index finger usually will have single tendon [16].

von Schroeder and Botte have reported two infrequent variant forms of extensor indicis proprius, namely, the extensor medii proprius (EMP) which gets inserted into the long finger and the extensor indicis et medii communis (EIMC) that splits into two tendons which get inserted into both index and long fingers [17]. The variant extensor indicis found in our case was almost similar to extensor indicis et medii communis mentioned above. A rare occurrence of EIMC together with anomalous extensor pollicis et indicis (EPI) muscle has been reported by Suwannakhan et al. [18]. The collective prevalence of EMP, EIMC, and EPI was reported to be 3.7, 1.6, and 0.75%, respectively [19].

Occurrence of hand extensor tendon variations cannot be ignored as surgeons must bear in mind the existence of these variations when performing common tendon transfers. It is also important to the orthopedic surgeons to ascertain the existence of additional tendons during the treatment of tenosynovitis.

## 4. Conclusion

The variations observed in our case with an anomalous superficial branch of radial artery at anatomical snuff box may be of significance in clinical point of view especially to vascular and plastic surgeons. The superficial arteries of the upper extremity may often be mistaken for veins, which may become a basis for intra-arterial injections instead of intended intravenous injections. Anatomic variation of extensor compartment of the hand may contribute to the development of tenosynovitis and limit the usefulness in the tendon transfer.
